# Don't Miss the Moment: A Systematic Review of Ecological Momentary Assessment in Suicide Research

**DOI:** 10.3389/fdgth.2022.876595

**Published:** 2022-05-06

**Authors:** Liia Kivelä, Willem A. J. van der Does, Harriëtte Riese, Niki Antypa

**Affiliations:** ^1^Department of Clinical Psychology, Institute of Psychology, Leiden University, Leiden, Netherlands; ^2^Leiden University Treatment Center LUBEC, Leiden, Netherlands; ^3^Department of Psychiatry, The Interdisciplinary Center Psychopathology and Emotion Regulation (ICPE), Universitair Medisch Centrum Groningen, University of Groningen, Groningen, Netherlands

**Keywords:** suicidal ideation, suicide attempt, experience sampling method, ambulatory assessment, electronic diary

## Abstract

Suicide and suicide-related behaviors are prevalent yet notoriously difficult to predict. Specifically, short-term predictors and correlates of suicide risk remain largely unknown. Ecological momentary assessment (EMA) may be used to assess how suicidal thoughts and behaviors (STBs) unfold in real-world contexts. We conducted a systematic literature review of EMA studies in suicide research to assess (1) how EMA has been utilized in the study of STBs (i.e., methodology, findings), and (2) the feasibility, validity and safety of EMA in the study of STBs. We identified 45 articles, detailing 23 studies. Studies mainly focused on examining how known longitudinal predictors of suicidal ideation perform within shorter (hourly, daily) time frames. Recent studies have explored the prospects of digital phenotyping of individuals with suicidal ideation. The results indicate that suicidal ideation fluctuates substantially over time (hours, days), and that individuals with higher mean ideation also have more fluctuations. Higher suicidal ideation instability may represent a phenotypic indicator for increased suicide risk. Few studies succeeded in establishing prospective predictors of suicidal ideation beyond prior ideation itself. Some studies show negative affect, hopelessness and burdensomeness to predict increased ideation within-day, and sleep characteristics to impact next-day ideation. The feasibility of EMA is encouraging: agreement to participate in EMA research was moderate to high (median = 77%), and compliance rates similar to those in other clinical samples (median response rate = 70%). More individuals reported suicidal ideation through EMA than traditional (retrospective) self-report measures. Regarding safety, no evidence was found of systematic reactivity of mood or suicidal ideation to repeated assessments of STBs. In conclusion, suicidal ideation can fluctuate substantially over short periods of time, and EMA is a suitable method for capturing these fluctuations. Some specific predictors of subsequent ideation have been identified, but these findings warrant further replication. While repeated EMA assessments do not appear to result in systematic reactivity in STBs, participant burden and safety remains a consideration when studying high-risk populations. Considerations for designing and reporting on EMA studies in suicide research are discussed.

## Introduction

Ecological momentary assessment (EMA) refers to data collection methods were momentary information is collected in real life (1). EMA is also known as experience sampling method (ESM) (2) or ambulatory assessment (AA) (3). These three terms emphasize the defining features of this methodology: catching individuals in their natural environments while they go about their daily lives, and probing them about their experiences as they unfold in the moment. Indeed, the most prominent strengths of EMA are its ecological validity and the ability to perform repeated assessments (1, 4). Technological advancements have further increased the feasibility of EMA measures: as opposed to undergoing assessments that are either based on retrospective self-report or performed in non-representative laboratory settings, participants may now provide time- and context-specific data through their smartphones (1, 3, 5).

While paper-and-pen diaries and later handheld computers or personal digital assistants (PDAs) were first used to collect EMA data, many studies now use mobile phone applications specifically designed for EMA purposes (6). These applications function as electronic diaries that may be used to prompt participants to record their mood, cognitions, behavior, context (incl. social interactions) and other experiences, typically either through text entries, event logs or rating scales (6). Such electronic EMA assessments typically use either *signal-contingent* or *event-contingent* sampling, prompting participants to fill out assessments either when alerted by the device, or when certain events naturally occur in their daily lives. These methods may also be combined (1, 6, 7). Signal-contingent sampling schedules can further be divided into *fixed* and *(pseudo)randomized* schedules. EMA assessments sent out on fixed schedules prompt participants at the same time(s) each day, while randomized schedules send out prompts at random times throughout the day; pseudorandomized schedules divide each 24-h period into blocks, and random prompts are sent out per block. Pseudorandomization offers advantages over full randomization, as it ensures that assessments are sufficiently paced out within the day, but also that participants do not systematically miss prompts due pre-determined commitments like work or school schedules, or learn to anticipate prompts (8).

EMA has been increasingly adopted in the study of psychopathology. This may be a promising approach since insights into the psychological states and behavior patterns in the daily life of the patient can be targeted in therapy (9). Recent reviews have outlined the applicability of EMA in a number of clinical populations, including patients with depression (10, 11) and anxiety disorders (12), eating disorders (13), borderline personality disorder (14), and psychotic disorders (15). These reviews indicate that EMA is an acceptable and feasible data collection method in psychiatric samples as well, and that it may be used to assess a range of experiences from affect (16) to self-harm (17) and substance use (18). Indeed, EMA can hold many advantages over traditional self-report measures for these purposes. Psychiatric disorders, such as depression (19, 20) and schizophrenia (21), are often characterized by memory biases. Retrospective accounts of certain behaviors, such as substance use, are also characteristically unreliable (8). Individuals may also be more willing to disclose sensitive information, such as accounts of drug use or self-harm, when they can do so remotely without face-to-face contact with the researcher (22). Further, EMA is an especially suitable method for assessing symptoms that are dynamic in nature (such as affective instability) (6, 23), which may be time or context dependent, and for which global retrospective measures provide only approximations (1). However, the benefits of EMA should be considered together with its possible limitations, which may include increased burden and time commitment from participants, and potential reactivity to repeated assessments of negative experiences (24).

Meanwhile, EMA remains a relatively underused data collection method in suicide research, although its features make it suitable for the assessment of suicidal thoughts and behaviors (STBs) (4, 25, 26). Suicide and suicide-related phenomena [ideation i.e., thoughts or fantasies about one's death (27), attempts] represent a major cause of mortality and disability worldwide (28, 29). Several risk-factors for suicide are known, including psychiatric and demographic variables such as depression, gender and stress (28–30). However, these factors have quite limited clinical use: they are poor predictors of short-term behavior, or are non-modifiable (e.g., gender, past STBs). Their base rate is also much higher than that of suicide, and basing clinical decisions on these risk factors would result in an abundance of false positives (31–33) and many interventions are generic and are not very efficacious (34). Meanwhile, acute warning signs of suicide risk remain less well studied and understood (35). Two recent meta-analyses concluded that there has been no improvement in the prediction of suicide risk in the past fifty years (31, 33). Many have called for a shift of focus towards prospectively predicting STBs in the short term (within days or even hours) (4, 34, 36). Both suicidal ideation and its risk factors can fluctuate substantially over short periods of time (days and hours) (37). Indeed, it has been suggested that (between-day) *variability* in suicidal ideation may be a better predictor of suicide than its intensity or duration (37, 38).

In summary, the study of STBs needs a new focus and methodology, for which EMA holds promise. Its limited use so far in suicide research may reflect concerns about the potentially adverse effects of repeated probing of suicidal thoughts and urges in at-risk groups. It has been demonstrated that asking individuals about their suicidal thoughts and behaviors does not induce suicidal ideation in asymptomatic individuals, nor does it increase risk in those affected. In fact, it may even serve to lessen ideation and general distress in high-risk individuals (39, 40). Limited evidence exists, however, on the question of whether this also holds for as frequently repeated assessments as with EMA schedules. The validity of EMA measures of STBs is also uncertain. Self-reports of suicidal behavior can be very unstable over time due to erroneous recall (41). Further, only a limited number of items can be used to cover a certain construct in EMA protocols (6)–sometimes only a single item is used [see e.g., (42)].

The aim of this systematic review was to determine: (i) how EMA has been used to operationalize and measure STBs (incl. methodology, aim, findings), and (ii) the feasibility, validity and safety of EMA in research on STBs. We exclude studies on non-suicidal self-injury (NSSI) [recently reviewed by (17)] and studies using paper-and-pen diaries, as these data are frequently compromised by retrospective responding (43).

## Methods

The review was conducted in accordance with the Preferred Reporting Items for Systematic reviews and Meta-Analyses (PRISMA) guidelines (44).

### Search Profile

**Figure 1:**
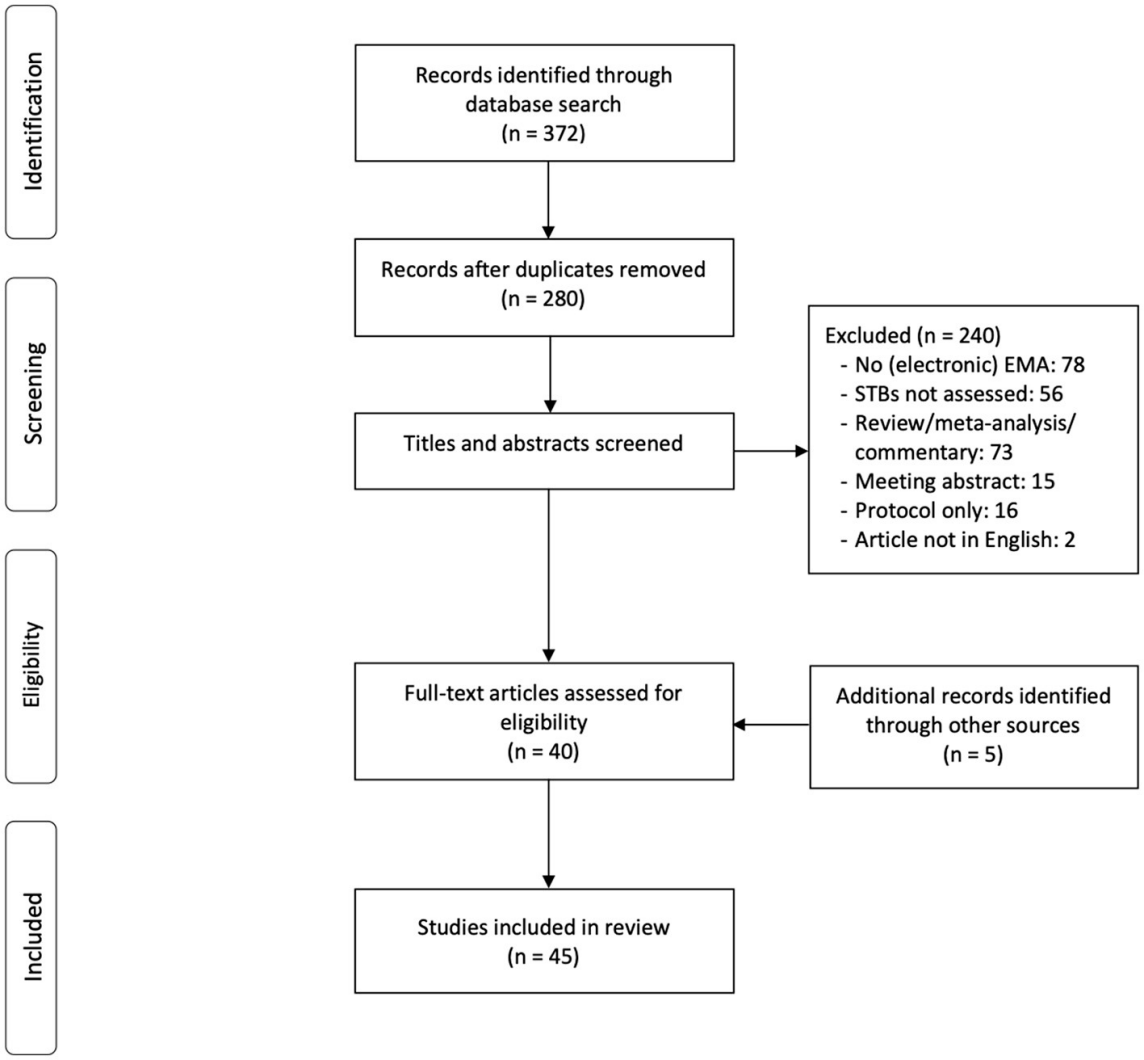
Preferred reporting items for systematic reviews and meta-analyses (PRISMA) flow diagram of included studies.

The databases Web of Science (www.webofknowledge.com) and PubMed (https://www.ncbi.nlm.nih.gov) were searched for articles in December 2021, using the search term: “((EMA) OR (“ecological momentary assessment”) OR (ESM) OR (“experience sampling method”) OR (“ambulatory assessment”) OR (“ambulatory monitoring”) OR (“real time monitoring”) OR (“electronic diary”)) AND ((“suicide”) OR (“suicidal”)).” As shown in Figure 1, the search produced 372 results. After excluding duplicate records, 280 remained. Of these, 40 met the inclusion criteria given below. Another 5 articles were identified through alternate sources (i.e., review papers and other articles), resulting in a total of 45 articles for the present review.

### Inclusion and Exclusion Criteria

We included articles reporting on (1) studies using electronic EMA (PDAs, mobile phones, smartwatches), and excluded studies using paper-and-pen diaries. We also included studies using web-based survey software (such as Qualtrics, www.qualtrics.com) if mobile phones or other devices were used to alert and direct the participants to the survey. We further only included (2) studies where EMA was used to assess STBs (≥1 item assessing STBs). We excluded studies focusing solely on non-suicidal self-injury (NSSI), but included studies where both NSSI and STBs were assessed.

Articles were also excluded if (1) the article was a meta-analysis, (systematic) review, editorial, or commentary, or (2) the article was not written in English.

### Data Abstraction

For each article we recorded the (1) author(s) and publication year, (2) sample characteristics, (3) aim of the study, (4) variable(s) measured through EMA, (5) how STBs were operationalized (i.e., the number and type of EMA items assessing STBs), (6) duration of the EMA assessment period, (7) sampling method (i.e., schedule and number of prompts per day), (8) device and software used, (9) methodological characteristics (incl. acceptance i.e., agreement to participate, attrition, compliance i.e., average response rates, and reactivity), and (10) main findings (as relating to STBs), including any adverse events. When reported, we also recorded any procedures used to ensure participant safety during the EMA assessment period.

## Results

In total, 45 articles reporting on 23 studies were included in the review (some studies were reported in more than one article; overlap between samples is indicated where applicable). Of these, 36 articles were reports where EMA was used to measure STBs (Table 1), and nine specifically addressed methodological issues (acceptability, feasibility and validity) of using EMA to measure STBs (Table 2).

**Table 1:** Overview of manuscripts reporting on studies using EMA to assess suicidal thoughts and behaviors (STBs).

**References**	**Sample**	**Aim**	**Measured variables**	**STBs items**	**Duration**	**Sampling method**	**Device**	**Compliance**	**Main findings**
Nock et al. (45)	*n* = 30 adolescents/ young adults w/ history of NSSI thoughts	Antecedents and functions of self-injurious thoughts and behaviors	Context, affect, coping, substance use, binging/ purging, STBs & NSSI thoughts and behaviors	1(−4) item(s): “Think of doing these? [ ] Attempt suicide.” (If *yes*: “How intense did the urge get?” “How long did you think about it?” “Did you attempt suicide?”)	14 days	(Fixed) signal-contingent 2x/day + event-contingent	Personal digital assistants (PDAs)	83% (filled in complete EMA)	SI most often occurred when alone, and was preceded by worry, feelings of pressure, bad memories, and arguments with others; SI was generally mild-to-moderate in intensity and longer in duration than NSSI thoughts
Ben-Zeev et al. (46)	*n* = 27 adult psychiatric inpatients	Real-time correlates of violent ideation and behavior	Context, affect, delusions, substance cravings/ withdrawal, violent ideation/behavior, SI	1 item: “Are you thinking of ending your life?”	7 days	Signal-contingent 6x/day	Customized Android smartphones	n/a	SI was associated with concurrent violent ideation and other-directed aggressive behavior
Husky et al. (42)	*n* = 42 adult recent suicide attempters	Predictors of SI in daily life	Activity, location, social interactions, stressful events, affect, negative thoughts (incl. SI and NSSI thoughts)	1 item: presence/absence of SI and/or NSSI	7 days	(Random) signal-contingent 5x/day	Personal digital assistants (PDAs)	74% (average response rate)	Inactivity, being at home/work, and feeling sad or anxious increased the probability of SI; being with close others decreased the probability of SI
Kleiman et al. (47) *Study 1*	*n* = 54 adult recent suicide attempters	Variability of SI and risk factors	Hopelessness, loneliness, burdensomeness, SI	3 items: “How intense is your desire to kill yourself right now?,” “How strong is your intention to kill yourself right now?,” “How strong is your ability to resists the urge to kill yourself right now?”	28 days	(Random) signal contingent 4x/day + event-contingent	Smartphones (mEMA software)	63% (average response rate)	Higher mean SI was associated with higher SI variability; hopelessness, loneliness and burdensomeness covaried with SI, but did not prospectively predict SI
*Study 2*	*n* = 36 adult psychiatric inpatients	Variability of SI and risk factors	Hopelessness, loneliness, SI	3 items: As above	Duration of inpatient stay (mean = 10 days)	(Random) signal contingent 4x/day + event-contingent	Android smartphones (MovisensXS software)	62% (average response rate)	Hopelessness and loneliness substantially (co)varied with SI, but did not prospectively predict SI
Hallensleben et al.* (48)	*n* = 20 adult psychiatric inpatients	Modeling variability of SI	SI	4 items: *Passive ideation* incl. “At the moment I feel that life is not worth living.” *Active ideation* incl. “At the moment I'm thinking about killing myself.”	6 days	(Random) signal-contingent 10x/day	Android smartphones (MovisensXS software)	n/a	SI variability was not significantly associated with baseline clinical characteristics (incl. depression severity)
Kleiman et al.** (49)	*n* = 43 adult recent suicide attempters	Affective antecedents and consequences of SI	Affect, SI	3 items: “How intense is your desire to kill yourself right now?,” “How strong is your intention to kill yourself right now?,” “How strong is your ability to resists the urge to kill yourself right now?”	28 days	(Random) signal-contingent 4x/day + event contingent	Smartphones (mEMA software)	n/a	NA decreased and PA increased at the next time point following instances of SI
Kleiman et al. (50) Study 1**	*n* = 51 adult recent suicide attempters	Phenotyping of suicidal ideators	SI	3 items: “How intense is your desire to kill yourself right now?,” “How strong is your intention to kill yourself right now?,” “How strong is your ability to resists the urge to kill yourself right now?”	28 days	(Random) signal- contingent 4x/day	Smartphones (mEMA software)	n/a	Five subtypes of SI: (1) low mean, low variability, (2) low mean, moderate variability, (3) moderate mean, high variability, (4) high mean, low variability, and (5) high mean, high variability
Study 2***	*n* = 32 adult psychiatric inpatients	Phenotyping of suicidal ideators	SI	3 items; As above	Duration of inpatient stay (mean = 9 days)	(Random) signal- contingent 4x/day	Android smartphones (MovisensXS software)	n/a	The finding of five subtypes of SI from Study 1 was replicated
Littlewood et al. (51)	*n* = 51 adults w/ current SI	Temporal relations of sleep and SI	Sleep, feelings of entrapment, SI	1 item: “Right now I am feeling suicidal.”	7 days	(Random) signal- contingent 6x/day	Smartwatch (PRO-Diary watch)	85% (average response rate)	Sleep duration, subjective sleep quality predicted next-day SI; daytime SI did not predict sleep the subsequent night
Coppersmith et al. ** (52)	*n* = 53 adult recent suicide attempters	Variability of SI and social support	Social support, SI	3 items: assessing (1) wish to live, (2) wish to die, and (3) desire to die by suicide, incl. “I have *no / a weak / a moderate to strong* wish to live.”	28 days	(Fixed) signal-contingent 1x/day	Smartphones (mEMA software)	71% (average response rate)	Perceived social support was negatively associated with same-day SI, but did not predict next-day SI
Czyz et al. (53)	*n* = 34 adolescents w/ a history of STBs	Proximal outcomes of a suicide intervention	Self-efficacy, safety plan use, coping, SI	1(−4) item(s): “At any point in the last 24h, did you have any thoughts of killing yourself?,” “How many times did you have thoughts of killing yourself?,” “How long did these thoughts last?,” “How strong was the urge to act on your thoughts of suicide?”	28 days	(Fixed) signal-contingent 1x/day	Text messages (TelEMA software) with link to online questionnaire (Qualtrics software)	n/a	Intervention group reported higher self-efficacy to resist urge to suicide, more sustained safety plan use, and more self-reliant coping
Czyz et al. **** (54)	*n* = 34 adolescents w/ a history of STBs	Co-occurrence and function of NSSI and SI	Coping, NSSI and SI	1(−4) item(s): As above	28 days	(Fixed) signal-contingent 1x/day	Text messages (TelEMA software) with link to online questionnaire (Qualtrics software)	n/a	SI and NSSI co-occurred on 58% of days, and on 98% of these days NSSI was reported as a coping mechanism for SI
Czyz et al. **** (55)	*n* = 34 adolescents w/ a history of STBs	Variability and predictors of daily SI	Hopelessness, connectedness, burdensomeness, SI	1(−4) item(s): As above	28 days	(Fixed) signal-contingent 1x/day	Text messages (TelEMA software) with link to online questionnaire (Qualtrics software)	69% (average response rate)	Connectedness, burdensomeness and loneliness were associated with same-day, but not next-day, SI
Hallensleben et al. (56)	*n* = 74 adult psychiatric inpatients	Variability and predictors of passive and active SI	Depressed mood, hopelessness, thwarted belongingness, burdensomeness, SI	4 items; *Passive ideation* incl. “Life is not worth living for me.,” “There are more reasons to die than to live.” *Active ideation* incl. “I think about taking my life.,” “I want to die.”	6 days	(Random) signal-contingent 10x/day	Android smartphones (MovisensXS software)	n/a	Passive and active SI associated with hopelessness, depressed mood, burdensomeness and thwarted belongingness; hopelessness and burdensomeness prospectively predicted SI at the next time point
Rath et al. * (57)	*n* = 74 adult psychiatric inpatients	Network modeling of SI and risk factors	Depressed mood, hopelessness, thwarted belongingness, burdensomeness, PA, anxiety, SI	4 items: “Life is not worth living for me.,” “There are more reasons to die than to live.,” “I think about taking my life.,” “I want to die.”	6 days	(Random) signal-contingent 10x/day	Android smartphones (MovisensXS software)	n/a	SI was concurrently associated with all risk factors; SI and perceived burdensomeness predicted SI at the subsequent time point
Rizk et al. (58)	*n* = 38 female adults w/ BPD and history of STBs	Variability of SI and its relation to affective instability	SI	9 items; assessing the wish to live, wish to die, wish to escape, thoughts about dying, thoughts about suicide, urge to die by suicide, thoughts about hurting self, urge to hurt self, and reasons for living	7 days	(Random) signal-contingent 6x/day	Personal digital assistants (PDAs)	n/a	Baseline affective instability predicted SI variability, independent of (baseline) depression severity
Spangenberg et al.* (59)	*n* = 74 adult psychiatric inpatients	Temporal stability of capability for suicide	Capability for suicide, SI	4 items (SI): “Life is not worth living for me.,” “There are more reasons to die than to live.,” “I think about taking my life.,” “I want to die.” 3 items (Capability for suicide): “Today I would have taken a lot of (physical) pain.,” “Today I was not at all afraid to die.,” “Today I could have killed myself if I wanted to.”	6 days	(Random) signal-contingent 10x/day + (Fixed) signal-contingent 1x/day	Android smartphones (MovisensXS software)	90% (random alerts), 95% (fixed alerts) (average response rate)	Substantial fluctuations in daily capability for suicide; daily SI was prospectively associated with suicide capability at the end of the day
Victor et al. (60)	*n* = 62 female adults w/ history of SI	Effects of internalizing and externalizing NA on SI	Internalizing & externalizing NA, rejection, criticism, SI and NSSI thoughts	1 item; “Since the last prompt, have you felt the urge or wanted to make a suicide attempt?”	21 days	(Random) signal-contingent 6x/day + (Fixed) signal-contingent 1x/day	Text messages with link to an online questionnaire	75% (average response rate)	Within-person changes in internalizing NA predicted SI at the next assessment; feelings of rejection and criticism were indirectly associated with SI through increased internalizing NA
Armey et al. (61)	*n* = 151 adults w/ history of STBs	Associations of SI, affect and anger	Affect, anger/ irritability, SI	1(−2) item: “Since your last completed questionnaire, have you thought about hurting or killing yourself?” (If *yes*, follow-up question on frequency)	21 days	(Random) signal-contingent 5x/day + event-contingent	Smartphones (mEMA software)	44% (average response rate)	Higher NA and lower PA associated with increased odds of SI; increased within-person anger/ irritability associated with increased odds of SI
Czyz et al. **** (62)	*n* = 32 adolescents w/ a history of STBs	Identifying early signs of suicide crises (attempt, hospitalization)	Self-efficacy, hopelessness, connectedness, burdensomeness, psychological pain, SI	1(−2) item(s): “At any point in the last 24 hr, did you have any thoughts of killing yourself?” (If *yes*: “How long did these thoughts last?”)	14 days	(Fixed) signal-contingent 1x/day	Text messages (TelEMA software) with link to online questionnaire (Qualtrics software)	76% (average response rate)	The strongest single predictors of suicide crises were duration of SI & self-efficacy
Hadzic et al.* (63)	*n* = 74 adult psychiatric inpatients	Association of trait impulsivity w/ variability in SI	SI	4 items: “Life is not worth living for me.,” “There are more reasons to die than to live.,” “I think about taking my life.,” “I want to die.”	6 days	(Random) signal-contingent 10x/day	Android smartphones (MovisensXS software)	n/a	Trait impulsivity associated with variability in passive, but not active, SI
Kaurin et al. (64)	*n* = 153 adults w/ BPD & n = 52 healthy controls	Associations of interpersonal stressors, affect, impulsivity and SI	Social interactions, affect, impulsivity, SI	2 items: “Have you wished you were dead or wished you could go to sleep and not wake up?,” “Have you actually had any thoughts of killing yourself?”	21 days	n/a	Smartphones (MetricWire application)	n/a	Interpersonal stressors associated with SI indirectly through higher NA and lower PA
Oquendo et al. (65)	*n* = 51 adults with MDD	Associations of affective instability, trait impulsivity and aggression, childhood trauma, stressful events & SI	Stressful evets, SI	9 items: “Thoughts about dying?,” “A wish to live?,” “A wish to die?,” “A wish to sleep and not wake up?,” “A wish to escape?,” “Reasons for living?,” “Thoughts about hurting yourself?,” “An urge to hurt yourself?,” “Thoughts about killing yourself?”	43 days (7 days at baseline, 3, 6, 12, 18, and 24 months follow-up)	(Random) signal-contingent 6x/day	Smartphones/ iPods (Harvest Your Data platform)	74%	High SI variability was associated with greater SI reactivity to stressors; degree of SI variability was stable over 24-months follow-up
Peters et al. (66)	*n* = 39 adult psychiatric inpatients	Correlates of SI variability	Depressed mood, anger/ irritability, social connectedness, SI	1 item: “How suicidal are you right now?”	Duration of inpatient stay (mean = 12 days)	(Fixed) signal-contingent 3x/day	Smartphones (Ethica platform)	Range 40-100% (daily average response rate)	SI severity and depressed mood variability were associated with greater SI variability, while general affective instability was not
Vine et al. (67)	*n* = 162 adolescents in current psychiatric treatment	Associations of dissociative experiences with SI	Dissociative experiences, affect, SI	2 items: “Thoughts about killing yourself or hurting yourself?,” “Told someone you were going to kill yourself or hurt yourself?”	4 days	(Fixed) signal-contingent 2x/day (weekdays) & 3x/day (weekends)	Smartphones	89% (reached target compliance rate of 80%)	SI was significantly associated with dissociative experiences, but only for female adolescents
Aadahl et al. (68) ******	*n* = 24 adults w/ current SI	Associations of metacognitive beliefs and SI	Defeat, entrapment, hopelessness, SI	2 items: “I want to die,” “I was thinking about killing myself”	6 days	(Random) signal-contingent 7x/day	Text messages with link to online questionnaire	49% (average response rate)	NA, hopelessness and defeat associated with SI
Al-Dajani et al. (69)	*n* = 39 community sample of adults w/ current SI	Function and consequences of SI	Affect, function of SI, SI	1(−3) item(s) (SI): “Since you last took this survey, did you experience a suicidal thought?” (If *yes*: “I thought that suicide can be a way to solve the problem I am facing.,” “Thinking of suicide was a way to escape/avoid the emotion I was feeling.”	14 days	(Random) signal-contingent 4x/day + event-contingent	Smartphones (Experience Sampler application)	68% (average response rate)	NA increased after instances of SI; seeing suicide as a solution (vs. escape) lead to a broader NA response following instances of SI
Cobo et al. (70)	*n* = 36 adult psychiatric patients w/ history of STBs	SI before and during COVID-19 lockdown	NA, sleep, appetite, SI	2 items: “Wish to die,” “Wish to live”	n/a	n/a	Smartphones (MEmind application)	n/a	SI (“Wish to die”) decreased during the COVID-19 lockdown
Hallard et al. (71)	*n* = 24 adults w/ current SI	Associations with cognitive control strategies, rumination and SI	Worry, rumination, self-punishment, distraction, social control, re-appraisal, SI	2 items: “I want to die,” “I was thinking about killing myself”	6 days	(Random) signal-contingent 7x/day	Text messages with link to online questionnaire	49% (average response rate)	Maladaptive cognitive control strategies (worry, punishment) and rumination associated with SI
Czyz et al. (72)	*n* = 74 adolescents w/ a history of STBs	Daily associations of NSSI and SI	NSSI and SI	1(−3) item(s): “At any point in the last 24 hr, did you have any thoughts of killing yourself?” (If *yes*: “How long did these thoughts last?,” “How strong was the urge to act on your thoughts of suicide?”)	28 days	(Fixed) signal-contingent 1x/day	Text messages with link to online questionnaire	74% (average response rate)	NSSI and SI co-occurred 78% of the time; longer and more intense SI increased the odds of engagement in NSSI; more engagement in NSSI was associated with higher odds of suicide attempt
Glenn et al. (73)	*n* = 48 adolescents	Short-term associations of negative interpersonal events and SI	Interpersonal events, thwarted belongingness and SI	4 items: “How intense is your desire to kill yourself right now?,” “How strong is your intent to kill yourself right now?,” “How able are you to keep yourself safe right now?,” “How strong is your desire to live right now?”	28 days	(Random) signal-contingent + interval-contingent 4x/day	Smartphones (mEMA software)	n/a	Thwarted belongingness mediated the association between negative interpersonal events and next-day SI
Kaurin et al. (74) *******	*n* = 153 adults w/ BPD & *n* = 52 healthy controls	Associations of sleep and next-day SI	Sleep, SI	6 items: “Have you wished you were dead or wished you could go to sleep and not wake up?,” “Have you actually had any thoughts of killing yourself?,” “Have you been thinking about how you might do this?,” “Have you had these thoughts and had some intention of acting on them?,” “Do you intend to carry out this plan?”	21 days	(Random) signal-contingent 6x/day	Smartphones (MetricWire application)	n/a	Increased sleep latency was associated with greater next-day SI
Porras-Segovia et al. (75) ********	*n* = 110 adult psychiatric patients w/ history of STBs	Associations of NA, appetite, sleep and SI	Sleep, appetite, NA, SI	2 items: “Wish to die,” “Wish to live”	Median 90 days	(Fixed & random) signal-contingent	Smartphones (MEmind application)	53%	Concurrent associations between disturbed sleep and SI
Schatten et al. (76) ****	*n* = 34 adolescent w/ a history of STBs	Affective predictors of same- and next-day suicidal ideation	Affect, SI	1 item: “At any point in the last 24h, did you have any thoughts of killing yourself?”	28 days	(Fixed) signal-contingent 1x/day	Text messages (TelEMA software) with link to online questionnaire (Qualtrics software)	69%	Misery, anger and happiness were associated with same-day SI; happiness predicted next-day SI
Stanley et al. (77) *****	*n* = 50 adults w/ BPD and history of STBs	Effectiveness of coping on SI	Coping, SI	9 items; assessing the wish to live, wish to die, wish to escape, thoughts about dying, thoughts about suicide, urge to die by suicide, thoughts about hurting self, urge to hurt self, and reasons for living	7 days	(Random) signal-contingent 6x/day	Personal digital assistants (PDAs)	70% (average response rate)	Distraction/positive activity-based coping strategies (e.g., keeping busy, socializing, doing something good for self) reduced intensity of SI at next time point
Victor et al. (78) *********	*n* = 161 female adults with history of SI	Associations between affect, NSSI and SI	Affect, NSSI, SI	1 item; “Since the last prompt, have you felt the urge or wanted to make a suicide attempt?”	21 days	(Random) signal-contingent 6x/day + (Fixed) signal-contingent 1x/day	Text messages with link to an online questionnaire	n/a	NA (mean and variability) was associated with SI
Wang et al. (79) ***	*n* = 83 adult psychiatric inpatients	Predicting suicide attempts from SI variability	SI	3 items; “How intense is your desire to kill yourself right now?,” “How strong is your intention to kill yourself right now?,” “How strong is your ability to resists the urge to kill yourself right now?”	Duration of inpatient stay (mean = 7 days)	(Random) signal-contingent 4–6x/day	Smartphones (movisensXS & Beiwe software)	n/a	Instability (rapid changes) in SI strongly predicted suicide attempt at 1-month follow-up

SI, Suicidal ideation; STBs, Suicidal thoughts and behaviors; NSSI, non-suicidal self-injury; BPD, borderline personality disorder; MDD, major depressive disorder; PA, Positive affect; NA, Negative affect; * sample from (56), ** (47) (Study 1), *** (47) (Study 2), **** (80) ***** (58), ****** (71), ******* (64), ******** (70), ********* (60).

**Table 2:** Overview of manuscripts assessing the feasibility and validity of using EMA to assess suicidal thoughts and behaviors (STBs).

**References**	**Sample**	**Aim**	**Measured variables**	**STBs items**	**Duration**	**Sampling method**	**Device**	**Main findings**
Husky et al.† (81)	*n* = 20 adult past suicide attempters, *n* = 42 recent suicide attempters, *n* = 13 healthy controls, *n* = 21 affective controls	Feasibility and validity of EMA in individuals at risk of suicide	Activity, location, social interactions, hopelessness, affect, (incl. SI & NSSI thoughts)	1 item: presence/absence of SI and/or NSSI thoughts	7 days	(Fixed) signal contingent 5x/day	Personal digital assistants (PDAs)	Acceptance: range 88% (recent suicide attempters)−67% (past suicide attempters), Compliance: range 86% (healthy controls)−74% (recent suicide attempters) (average response rate), Reactivity: no effect of study duration on intensity or duration of NA & no effect on frequency of SI, Validity: baseline depression scores predicted EMA NA (incl. SI)
Law et al. (82)	Adults w/ and w/o BPD: *n* = 119 control EMA and *n* = 129 EMA w/ SI items	Reactive effects of repeated assessment of STBs	BPD symptoms, affect, STBs	2 items: “I tried to kill myself in the last 60 min.,” “I thought about committing suicide in the last 60 min.”	14 days	(Fixed) signal-contingent 5x/day	Personal digital assistants (PDAs)	Retention: 96%, Compliance: 78% (suicide EMA) vs. 80% (control EMA) (average response rate), Reactivity: No reactive effects of repeated assessment of STBs on the occurrence of SI, self-harm or suicide attempts for either BPD or non-BPD sample
Torous et al. (83)	*n* = 13 adults w/ MDD	Feasibility and validity of EMA for depressive symptoms	Depressive symptoms (PHQ-9)	1 item: “I would be better off dead or hurting myself.”	30 days	(Random) signal-contingent 3x/day	Smartphones (Mindful Moods app)	Acceptance: 93%, Compliance: 78% (average response rate), Validity: EMA depression scores (incl. SI) correlated highly with the PHQ-9 (*r* = 0.84), but more SI was reported through EMA
Czyz et al. †† (80)	*n* = 34 adolescents w/ history of STBs	Feasibility of using EMA in adolescents at risk of suicide	STBs, experience with EMA	2(−6) item(s): “At any point in the last 24 h did you have any thoughts of killing yourself?” (If *yes*: “How many times did you have thoughts of killing yourself?,” “How long did these thoughts last?”), “At any point in the last 24 h, did you try to kill yourself or make yourself not alive anymore?” (If *yes*: “What did you do?,” “Did you do this as a way of ending your life?”)	28 days	(Fixed) signal-contingent 1x/day	Text messages (TelEMA software) with link to online questionnaire (Qualtrics software)	Acceptance: 77%, Retention: 69%, Compliance: Average 69% (Week 1: 80%, Week 4: 60%) (average response rate), Validity: SI endorsed by 71% in EMA vs. 45% in retrospective interview
Forkmann et al. ††† (84)	*n* = 74 adult psychiatric inpatients w/ depression and history of SI	Psychometric properties of EMA SI items	PA, anxiety, depression, burdensomeness, thwarted belongingness, hopelessness, SI	4 items: *Passive ideation* incl. “At the moment I feel that life is not worth living.” *Active ideation* incl. “At the moment I'm thinking about killing myself.”	6 days	(Random) signal-contingent 10x/day	Android smartphones (MovisensXS software)	Acceptance: 47%, Retention: 94%, Compliance: 90% (average response rate), Validity: EMA SI correlated strongly with retrospective questionnaire (BSSI) (Passive SI: *r* = 0.73, Active SI: *r* = 0.76), Reliability: Passive SI: α = 0.80, Active SI: α = 0.79 (within-person), Passive SI: α = 0.97, Active SI: α = 0.92 (between-person)
Glenn et al. †††††† (85)	*n* = 53 adolescents and their parents	Feasibility and acceptability of EMA in high risk adolescents	Sleep, affect, cognitions, substance use, interpersonal negative events, SI	6 items: “How intense is your desire to die right now?,” “How strong is your intent to kill yourself right now?,” “How able are you to keep yourself safe right now?,” “Are you thinking about attempting suicide (hurting yourself to die)?,” “Did you do anything to hurt yourself (with or without wanting to die) today?” (If *yes*, follow-up questions on intensity and duration)	28 days	(Random) signal-contingent + interval-contingent 5x/day	Smartphones (mEMA software)	Acceptance: 25%, Compliance: Average 63% (Week 1: 87%, Week 4: 45%) (average response rate)
Gratch et al. †††† (86)	*n* = 51 adults with MDD	Validity of EMA-assessed SI	SI	9 items: “Thoughts about dying?,” “A wish to live?,” “A wish to die?,” “A wish to sleep and not wake up?,” “A wish to escape?,” “Reasons for living?,” “Thoughts about hurting yourself?,” “An urge to hurt yourself?,” “Thoughts about killing yourself?”	7 days	(Random) signal-contingent 6x/day	Smartphones/iPods (Harvest Your Data platform)	Compliance: 73% (average response rate), Validity: Worst point EMA SI correlated with retrospective questionnaire (BSSI; *r* = 0.73); 58% reporting SI in EMA did not do so on the BSSI
Porras-Segovia et al. ††††† (87)	*n* = 120 adult psychiatric patients w/ history of STBs & *n* = 337 student controls	Feasibility of EMA in psychiatric patients and controls	NA, sleep, appetite, SI	2 items: “Wish to die,” “Wish to live”	60 days	n/a	Smartphones (MEmind application)	Acceptance: 64% psychiatric patients vs. 69% controls, Retention: 68% (controls) vs. 80% (psychiatric patients), Compliance: 65% psychiatric patients vs. 75% controls (average response rate)
Rogers et al. (88)	*n* = 237 community sample of adults w/ current SI	Feasibility and acceptability of EMA in a high-risk community sample	Affect, hopelessness, loneliness, agitation, irritability, rumination, thwarted belongingness, social interactions, stressful events, sleep, SI	Incl. SI thoughts, intent & desire, suicide plans, preparations, attempt	14 days	(Random) signal-contingent 6x/day	Smartphones (Ethica platform)	Compliance: 69% (average response rate), Retention: 60%

*SI, Suicidal ideation; STBs, Suicidal thoughts and behaviors; NSSI, non-suicidal self-injury; BPD, borderline personality disorder; MDD, major depressive disorder; PA, Positive affect; NA, Negative affect; PHQ-9, the Patient Health Questionnaire-9; BSSI, Beck Suicide Severity Index; †sample corresponds to (42), †† (53), ††† (56), †††† (65), ††††† (70), †††††† (73) reported in Table 1*.

### Characteristics of EMA Studies Measuring STB's

#### Samples

Sample sizes ranged from 13 to 457 (median = 53, *n* = 23). Most studies (78%, *n* = 18) were conducted in adult, and less frequently in adolescent samples [22%, *n* = 5; (45, 67, 72, 80, 85)]. Participants were typically recruited from high-risk populations, such as psychiatric inpatients or those recently discharged from the hospital. Most frequent primary co-morbid diagnoses were depressive disorders (83, 84, 86) and borderline personality disorder [BPD (58, 64, 82)]; however, inclusion was typically based on (recent) history of self-reported STBs to ensure sufficient number of observations of STBs during the assessment period.

#### Schedules

The duration of EMA monitoring ranged from 4 to 60 days (median = 14, *n* = 23). The number of (scheduled) EMA prompts per day ranged from 1 to 11 (median = 5, *n* = 21). All studies used some form of signal-contingent sampling: (pseudo)random sampling schedules were most frequently used [57%, *n* = 13 (42, 47, 51, 58, 61, 65, 69, 71, 79, 83, 85, 86, 88)], followed by fixed sampling [26%, *n* = 6 (45, 66, 67, 72, 80, 82)], and protocols that combined both fixed and (pseudo)random sampling [13%, *n* = 3 (47, 56, 60)]. Fixed schedules were almost exclusively used in studies with once-daily prompts (as well as three older studies with PDAs (45, 81, 82)), whereas pseudo-random schedules were typically used for repeated within-day assessments. Approximately one fourth (26%; *n* = 6) of studies supplemented signal-contingent sampling with event-contingent sampling [i.e., participants were encouraged to self-initiate additional entries when experiencing STBs (45, 47, 61, 69, 85)], but none of the studies used event-contingent sampling alone. Studies frequently (57%, *n* = 13; (47, 51, 58, 60, 61, 69, 71, 79–81, 85, 86)) reported that participants could provide input about their daily schedules (incl. sleep and wake times), allowing EMA prompt windows to be adjusted for each participant, and a minimum time window (30–60 min) between prompts was established with (pseudo)random schedules to achieve better temporal coverage.

#### Measured Variables and Operationalization of STBs

While all studies included EMA items on suicidal ideation, four studies (18%) also assessed the occurrence of suicide attempts via EMA (45, 80, 82, 88) (see Tables 1, 2 for full list of measured variables and SI item descriptions). The number of EMA items on STBs ranged from 1 to 9 (median = 2, *n* = 22). The items were typically rated on a 5-point Likert-scale; seven (32%) studies used binary items, or a combination of an initial binary item on the presence of STBs, followed by ratings on frequency, intensity and/or duration (18%, *n* = 4). Items were often based on established self-report questionnaires or structured interviews, such as the Beck Scale for Suicide Ideation (BSSI) (89) or the Columbia Suicide Severity Rating Scale (C-SSRS) (90), and rephrased to reflect the time period of the EMA or otherwise adapted for the purposes of the study.

Several studies used gate questions to limit the number of questions presented pertaining to STBs. Such gate questions either first inquired about the presence of (any) negative thoughts prior to direct questioning of suicidal ideation [see e.g., (81)], or limited follow-up questions on the intensity, frequency and/or duration of ideation only to those instances where suicidal ideation was first endorsed [see e.g., (45, 55, 61, 85)]. Two studies used a turn-over system where a subset of questions was randomly presented at a certain time point to limit repetition (83, 87). Studies were heterogenous in their operationalization of STBs, and no clear delineation emerged over time on preferred methodologies or use of specific EMA items.

The most frequently measured predictor variables included contextual factors (incl. location, activity, social company), affect, and constructs from the Interpersonal Psychological Theory of Suicide [IPTS: hopelessness, burdensomeness and thwarted belongingness (91)]. Protective factors, such as coping and social support, were less frequently assessed.

### Main Findings

#### Prevalence of STBs

In adolescent samples, suicidal ideation was reported by 34–82% of the sample during EMA (median = 71%, *n* = 3), and overall, 2–39% of observations had suicidal ideation ratings >0 (median: 25% *n* = 3). These thoughts occurred once a week on average, and typically lasted 1 to 30 min [based on a binary measure of ideation (45)]. In adult samples, ideation was reported by 26–100% of the participants (median = 97%, *n* = 7), and 1–82% of observations had suicidal ideation ratings > 0 (median: 22% *n* = 7). While the majority of studies recruited participants with heightened risk profiles (such as those recently discharged after a suicide attempt), prevalence rates in two community-based samples with current self-reported ideation were comparable to the pooled prevalence rates (86–100% participants and 20–22% of all entries indicated suicidal ideation (69, 88). When examined separately, higher levels of passive (*m* = 4.54, *sd* = 2.25, range 2–10) than active (*m* = 3.18, *sd* = 1.50, range 2–10) suicidal ideation was reported (56).

Contextual factors of suicidal thoughts among adolescents included being alone, experiencing arguments/conflict or recalling negative memories (45). Among adolescents with a history of NSSI, suicidal ideation frequently co-occurred with NSSI (72). Among adults, being alone, at home or at work, and inactivity increased the probability of suicidal ideation, while being with family and friends or engaged in leisure activities decreased probability of ideation (42). Although negative daily life events were generally not associated with suicidal ideation, negative interpersonal events increased the probability of ideation (42, 64), whereas perceived social support decreased its probability (52). Affective precipitants (incl. negative affect, feelings of pressure, anger/irritability) were associated with increased occurrence of ideation (45, 61).

#### Variability of STBs

Most individuals experienced substantial variability in suicidal ideation both between- (55) and within-days (47, 56, 58). Within-day, approximately one third of ratings differed from the previous one by at least one (within-person) standard deviation, illustrating both sharp increases and decreases in ideation in a time frame of hours [4–8 h (47)]. Those with higher mean ideation (per person, across EMA period) experienced more variability (47, 65, 66). Risk factors (negative affect, hopelessness, loneliness, burdensomeness, connectedness, thwarted belongingness) occurred with similar variability, and were concurrently associated with suicidal ideation (47, 55, 56, 68, 78). General affective instability (i.e., tendency to experience frequent, sudden changes in mood) was associated with suicidal ideation variability among female BPD patients (58), and inpatient individuals diagnosed with MDD or bipolar disorder (66). Generally, baseline clinical characteristics, such as severity of depressive symptoms [retrospective self-report of symptoms over the past 2 weeks (48)] were not differentially associated with suicidal ideation variability. The test-retest reliability of EMA-assessed within-person suicidal ideation variability (as estimated by the Root Mean Square of the Successive Differences, RMSSD) was high across 24 months (65). Suicidal ideation variability (here operationalized as the individual's likelihood of experiencing extreme changes in suicidal ideation from one assessment point to the next) was also predictive of the occurrence of a suicide attempt at 1-month follow-up post-discharge, based on a pilot study of 83 adults hospitalized for a suicidal crisis (79).

#### Prediction of STBs

Most reports failed to establish independent temporal predictors of suicidal ideation severity: of twelve articles fitting temporal prediction models (47, 51, 52, 55–57, 60, 73, 74, 76, 77), four failed to establish significant predictors after accounting for ideation at the previous time point (47, 52, 55), and five did not control for prior ideation (51, 60, 73, 74, 76). Across studies, prior suicidal ideation therefore remained the strongest (or only) predictor of subsequent ideation (i.e., suicidal ideation at T significantly predicting ideation at T+1). Regarding other predictors, the most consistent evidence was found for momentary negative affect, hopelessness and burdensomeness. These variables predicted increased momentary suicidal ideation within-day (47, 56, 57, 60). One study indicated that active coping reduced the intensity of ideation at the subsequent assessment 2 h later (77). Between days, short sleep duration (both objective and subjective), poor subjective sleep quality and increased sleep latency (i.e., time to fall asleep) predicted (mean) next-day suicidal ideation (51, 74). Negative interpersonal events were also associated with increased next-day suicidal ideation (73). The probability of finding influential predictors was further lower with increasing intervals. Studies examining day-to-day rather than within-day changes in suicidal ideation were less likely to report positive findings (55, 80). This may be due to reduced temporal granularity of data due to aggregate daily ratings.

### The Methodology of Using EMA to Assess STBs

In order to examine the feasibility of using EMA in suicide research we reviewed reports of acceptance and compliance across studies, as well as detail previously used measures to ensure participant safety during EMA periods. Reports of adverse events are further examined to estimate the safety of repeated assessments of STBs.

#### Acceptance and Compliance

Acceptance rates ranged between 25 and 93% (median = 77%, *n* = 10). Comparing three subgroups, acceptance was highest among outpatients with a recent history of a suicide attempt (88%), as compared to clinical controls (i.e., 68% outpatients without a history of suicide attempts), and healthy controls [77%; (81)]. Acceptance was lower in inpatient samples (47–77%, median = 50%, *n* = 3).

Compliance ranged from 44 to 90% (median 70%, *n* = 19). Compliance in clinical subgroups (range 74–82%) was lower than that in a non-clinical control group (86%) (81). A similar pattern emerged when comparing psychiatric patients (65%) and student controls (75%) (87). Compliance rates were not significantly related to suicide history or current depressive symptom or suicidal ideation severity (65, 66, 71, 85, 88). Compliance rates declined over time (i.e., participants exhibited fatigue effects) (80, 84, 85). In a 4-week study, compliance decreased by twenty percentage points from the first to the fourth week of EMA assessment (80). However, this effect was not replicated by all: rather than declining in a linear manner, one study reported that compliance rates did not decrease over time (66), fluctuated before stabilizing after ~2 weeks (83), or that compliance increased over time during a 1-week EMA study (81). Compliance rates did not differ between studies employing once-daily (range 69–74%, median = 72%, *n* = 2), or multiple daily assessments (range 44–90%, median = 70%, *n* = 17). Response rates were higher in the afternoons (83) and on weekend days (84). Practice effects were also observed by participant's response times decreasing over time (81).

Attrition was low (4–40%, median = 6%, *n* = 10). In contrast to findings of lower compliance rates among psychiatric patients, dropout was lower among clinical cases than controls (87). The highest attrition rate (40%) was reported in an anonymous online study with no personal contact (88).

#### Validity

EMA measures were associated with traditional self-report and interview measures. Baseline depression severity [Hamilton Depression Rating Scale, HAMD (92)] predicted EMA-assessed sad mood and negative thoughts (incl. suicidal ideation) (81). The correlation between depression scores (incl. a suicidal ideation item) derived from the traditionally administered Patient Health Questionnaire-9 [PHQ-9 (93)] and EMA administered PHQ-9 was *r* = 0.84 (83). EMA-measured momentary suicidal ideation correlated highly^1^ with the BSSI [passive ideation: *r* = 0.73, active ideation: *r* = 0.76 (67)]. Correlations were higher for items assessing active (“Wish to die” *r* = 0.76) rather than passive ideation (“Wish to live” *r* = 0.37) (86). A one-item EMA measure (“How suicidal are you right now?”) correlated highly with the BSSI (*r* = 0.71) and moderately with the Beck Depression Inventory [BDI (95)] [*r* = 0.41 (71)]. Variability in momentary SI correlated moderately with the Suicide Behaviors Questionnaire - Revised [SBQ-R (96)] (*r* = 0.41), the BSSI (*r* = 0.49), and the Capability for Suicide Questionnaire [GCSQ (97)] (*r* = 0.30) (63).

More severe depressive symptoms were reported through EMA than with a traditional retrospective questionnaire, and EMA reports of suicidal ideation were notably higher than questionnaire scores for 69% of the participants (83). In an adolescent sample, suicidal ideation was reported in EMA by 71% of the participants, and in 45% of the interviews post-EMA (80). Among adults, 58% of participant reporting SI in EMA did not do so in an interview post-EMA (86).

#### Reactivity in Momentary Affect and STBs

A feasibility study in adult suicide attempters (recent or past attempt history), clinical controls (i.e., depressed patients without suicide attempt history), and healthy controls, found no effects of study duration on the intensity of negative affect or frequency of suicidal ideation, indicating no symptom worsening with repeated prompts (81). However, there was a decrease in positive affect among recent and past suicide attempters, *and* a decrease in hopelessness among recent suicide attempters with increasing study duration (across seven days) (81). In another study comparing two 14-day EMA protocols (one with items on suicidal ideation, and a control EMA protocol), there were no differences in the occurrence of suicidal ideation, self-harm or suicide attempts between the two conditions for either clinical (patients with BPD) or non-clinical controls based on weekly retrospective measures (82). In a sample of adolescents assessed after 1-month of EMA, most participants reported that they generally felt no change in mood after filling out EMA (69%) or that they felt better (28%); one participant reported that they had worse mood after completing EMA (80). The clinicians of another adolescent sample reported the study, on average, to have had “neutral” to “somewhat positive” impact on their patients [incl. increased awareness into one's condition (72)]. Following a 6-day EMA assessment with 10 prompts per day, 16% of a sample of depressed inpatients reported that they had felt stressed and/or burdened by the assessments (84), but no further details were provided. Among 237 high risk adults from the community, 9% reported they had experienced the EMA as “occasionally' distressing,”' “emotionally taxing,” and, “triggering bad thoughts,” (p. 6) in comparison to 3% who reported a decrease in the frequency of and urge to act on suicidal thoughts due to study participation (88). In general, participants reported their experiences overall as neutral-to-positive but time consuming (or burdensome), and that they would be open to participating in similar research in the future (80, 84, 85).

#### Adverse Events

Ten studies reported whether any suicide attempts occurred during the study period: in four studies no such events occurred (45, 47, 84). Three studies followed adolescents who were recently discharged from inpatient treatment after a suicide attempt or severe ideation. In 28 days, the incidence of suicide attempts was 6% (55), 8% (72), and 9% (85). In a sample of 50 adult BPD patients, 10% attempted suicide over 7 days (77), and in a study of 248 adults with and without BPD, approximately 5% of participants made a suicide attempt during the *entire* study period (including a 6-month follow-up) (82). In a community sample of 237 adults with current suicidal ideation, 3% attempted suicide during the 2-week study (88). In comparison, in similar high-risk populations (with last-year suicidal ideation or attempt) the estimated 1-year prevalence of suicide attempts is between 13 and 20% (98, 99), with the risk being higher for those with recent attempt history (99). Risk is further heightened among those with an earlier age of occurrence of first attempt, as well as those with borderline personality disorder (features) (100). No suicide mortality was reported in any of the reviewed studies.

#### Safety Measures

Eight studies reported implementing some type of safety measures in their EMA protocols. Four studies implemented automatic messages sent out by the EMA device. In one study each EMA assessment began with a message reminding the participant to contact a mental health professional or emergency personnel in case of a crisis (82), and three others used similar messages that were presented if the participant's responses indicated momentary suicidal ideation (42, 61, 80). Three studies employed ongoing monitoring of the participant's responses (45, 72, 80). In a study using PDAs, participants were instructed to upload their data on a server each night for evaluation, and research personnel phoned participants in case responses indicated imminent risk or if no data had been uploaded for 72 h (45). Another study reported twice-daily (manual) checks on the participants entries; 32% of the adolescent participants were contacted for a risk assessment during the 4-week study (72). In another study, the EMA software was programmed to send out automatic email alerts to the study's on-call clinician if the participant endorsed a suicide attempt or severe ideation with suicidal intent and/or a plan, in which case the clinician made contact with the participant; <1% of the responses recorded met this threshold and required contact by the study personnel (80). Two studies required that each participant had an individualized safety plans in place established by their treating physician (61, 85), and another study instructed participants on how to make one prior to participation (88). In two studies, research personnel conducted an unspecified suicide risk assessment halfway through the 2-week EMA period (69), and in the other study participants completed the CSSRS at baseline and at follow-up and test assistants referred acute cases to the emergency department (70). Of note is that while only 36 % (*n* = 8) of studies reported on safety procedures, 80% (*n* = 4) of studies in adolescent samples had safety measures in place. None of the studies conducted in inpatient settings employed additional safety measures.

## Discussion

### Applicability of EMA in Suicide Research

Among the 23 reviewed studies, substantial variability existed in the operationalization of STBs. This ranged from single-item binary measures of general self-harm ideation (42) to multi-item batteries assessing the intensity, frequency and duration of specific suicidal thoughts [see e.g., (55, 65)]. General guidelines for EMA research emphasize that items should be formulated in a way that allows for the assessment of the natural fluctuations in momentary experience, while limiting potential floor and ceiling effects (101). Binary items generally lack these characteristics. Single-item measures may also not be sufficient in capturing the wide spectrum of ideation, such as distinguishing passive from active ideation and intent. Further, suicidal ideation alone is not the only permissive characteristic preceding suicidal acts; a transition from ideation to attempt requires *acquired capability*, that is, additional cognitive and behavioral processes, such as decreased fear of death and increased pain tolerance (30). These latter characteristics can also fluctuate substantially from day to day (59).

The strength of EMA for suicide research remains in its ability to capture more variable aspects of suicide risk that may be difficult to grasp by traditional retrospective questionnaires. From our review we conclude that suicidal ideation exhibits substantial variability over time, often increasing or decreasing sharply within only a few hours in an individual [see e.g., (47)]. Witte and colleagues (37, 38) have proposed that such variability in suicidal ideation may provide a more reliable index of suicide risk than the severity or duration of ideation alone. This notion is tentatively supported by findings of higher suicidal ideation variability among patients with more severe suicidal ideation (47, 65, 66), as well as those with a prior suicide attempt history (66), and by higher EMA suicidal ideation variability predicting attempts at 1-month follow-up (79). In line with these findings, a previous review of EMA studies on NSSI also identified affective variability as a risk factor for engaging in self-harm behavior (17). While these preliminary findings warrant further replication, they indicate that suicidal ideation variability may represent a promising marker for suicide risk.

In addition to suicidal ideation itself, a number of its risk factors (incl. negative affect, hopelessness, loneliness, burdensomeness, thwarted belongingness) were also found to exhibit similar variability patterns and associate with momentary ideation. However, fewer studies so far have succeeded in establishing prospective predictors of suicidal ideation. A similar pattern is observable in the EMA literature on NSSI, where most studies have elucidated on the immediate context, rather than precipitants, of self-harm behavior (17). Kaurin et al. (64) outlined the ongoing discourse in EMA literature over the relative value of time-lagged vs. concurrent (or *contemporaneous*) modeling approaches. While longitudinal modeling is often regarded as superior in traditional research designs, contemporaneous associations derived from EMA data reflect associations beyond simple co-occurrences; rather, they reflect systematic covariances between variables, and can signal the presence of temporal associations occurring very close in time. Hence, these findings indicate that a number of known longitudinal predictors of suicidal ideation are also involved in its imminent emergence over shorter time frames. Considering emerging evidence that suicidal ideation variability may represent an important marker for acute risk, increased understanding of the factors underlying these fluctuations is of great importance.

### Feasibility and Safety of EMA in Suicide Research

#### Acceptability and Compliance

While our review supports the general acceptability of EMA in suicide research, the burden of EMA measures may be less tolerable for those currently experiencing very severe symptoms, analog to findings in individuals with depressive disorders (102). Meanwhile, compliance was good and not substantially lower than in other clinical (103) or non-clinical populations [see e.g., (104)]. This is in line with reports that EMA compliance is not significantly influenced by demographic or clinical characteristics (105).

Regardless, maintaining compliance with EMA remains a challenge, especially when assessment periods grow long, as compliance decreases over time with each subsequent week of EMA [see e.g., (80, 85)]. Meanwhile, compliance rates did not appear lower in studies using multiple measures per day (vs. once-daily ratings). It has also previously been reported that more frequent assessments may not reduce compliance (106), or may even increase compliance (107), as long as questionnaires are kept brief (108). Shorter time intervals between prompts can also increase compliance (109). However, overly lengthy measures can induce fatigue and reduce compliance, as well as impact data quality due to increased careless responding or skipping questions (108, 110). Based on our review, researchers may be advised to prioritize more frequent, but brief assessments over short time periods to establish higher compliance; future research should aim to more systematically examine how increasing the number of daily prompts affects compliance rates, in order to establish optimal sampling schedules that balance temporal coverage with participant burden. Researchers may also consider implementing incentives for compliance. Many of the reviewed studies used monetary rewards for increasing or sustaining compliance [see e.g., (85, 88)]. However, monetary incentives are reported as relatively unimpactful in increasing compliance, based on a review of 481 EMA studies (111). Alternative incentives, such as personalized feedback based on EMA data, may be regarded as more valuable (112).

In line with the observation that all of the reviewed studies used signal-contingent sampling (either alone or in conjunction with event-contingent sampling), we may also recommend this approach for future research, as signal-contingent sampling more optimally allows for the examination of the variability in experience of STBs. Finally, further research is needed to generalize these recommendations to other age groups (such as the elderly) and non-Western societies. As the reviewed studies exclusively focused on adolescents and adults (who may already be more accustomed to using technology to track their lives), it remains to be established whether such electronic symptom self-monitoring would be perceived as equally acceptable, and helpful, by older populations.

#### Validity

While EMA measures showed high correlations with traditional self-report, more individuals reported suicidal ideation through EMA, and more severe instances of ideation were detected through EMA than retrospective measures. We further found that EMA reports of active suicidal ideation were more highly correlated with retrospective measures than those of passive ideation (86). It is tempting to speculate that EMA has increased sensitivity in detecting momentary, fleeting, and/or passive instances of ideation. However, the possibility that part of this increased reporting is due to reactivity to the EMA questions (i.e., symptom increases due to enhanced focus on them) cannot be disregarded (24, 113), although the current evidence does not support such assessment reactivity (see below).

#### Adverse Events

Our review did not uncover systematic (negative) mood reactivity to EMA, and importantly, there was no evidence of reactivity on STBs specifically (81, 82). These findings are in line with reports of no symptom reactivity in other patient populations, such as those with chronic pain (114) and mood disorders (81). Some behaviors, like alcohol use among substance dependent patients, may be more subject to reactive effects than cognitive or affective symptoms (103). However, these conclusions are tentative at best due to the low number of studies directly assessing reactivity, and the general lack of control groups across studies. Further, available studies were seriously limited in their assessment and reporting of adverse events (suicide attempts, mortality) occurring during the study period. Future research should more transparently examine and describe these events if, and when, they occur.

#### Safety Considerations

A defining strength of smartphone-based EMA for suicide research is that it enables the real-time monitoring of participant's responses. However, it remains to be determined how such risk detection can be done with optimal sensitivity and specificity. Changes in symptoms over time, especially drastic changes over short periods of time (within days, hours), may provide a better indication of risk than absolute ratings at any single time point (35). Further, participants may not always provide accurate reports of their experiences for fear of intervention, as many people planning suicide explicitly deny such intentions (115). EMA safety protocols should consequently also involve contact with participants lost to attrition, and additional contact should be made not only when participants indicate severe symptomatology, but also when EMA prompts are systematically missed [as also previously done by e.g., (45)].

### Limitations

Across the reviewed studies, there was considerable heterogeneity in study characteristics and their reporting thereof. This, together with the diversity in aims and samples across studies, prevented us from conducting meta-analyses. Little rationale was provided for the selection of the EMA items used (or if pilots were run to establish the item set for the population under study) with the exception of questions adapted from established self-report questionnaires. However, these questions may not always optimally translate to EMA, as they can lack sufficient sensitivity to variability, especially over shorted time frames. Notably, three (14%) studies did not provide EMA item descriptions, two (9%) did not report sampling frequency, and three (14%) did not report sampling technique (i.e., fixed or random). Further, there was insufficient reporting of other study characteristics: 12 (55%) studies did not report acceptability, three (14%) did not report any index of compliance (with further inconsistencies in how compliance was defined), 14 (63%) did not report on attrition, 12 (55%) did not report adverse events, and 11 (50%) did not report whether any safety measures were implemented. Additional characteristics that may impact data quality and inference, such as amount and patterns of missing data, and information on average time intervals between prompts, as well as delay from alert to response, were rarely reported. A recent review of EMA of NSSI noted similar study heterogeneity and lack of reporting on compliance (17). Reviewers evaluating EMA studies for publication should require these to be reported. Finally, how to adequately measure EMA item reliability and validity remains to be established (although first initiatives have started, such as the Experience Sampling Method (ESM) Item Repository (https://osf.io/kg376/). Correlations with retrospective measures, or moment-to-moment reliability statistics may not provide adequate indications of good psychometric fit, as EMA ratings are *expected* to vary over time rather than stay constant.

### Future Directions

Based on the reviewed studies, in Table 3 we provide an overview of considerations for designing and reporting on EMA studies in suicide research. Directions for future research are discussed further below.

**Table 3:** Considerations for designing and reporting EMA studies in suicide research.

**Design**
1. Manage burden	Assessments should be quick and easy to complete in daily life. More frequent prompts over shorter time periods do not necessarily reduce compliance, while longer assessment periods may. Feedback from participants over preferred sampling windows may reduce the burden of ill-timed prompts and increase compliance.
2. Sensitivity to change	EMA items should be able to capture (more fine-tuned) changes in symptoms over time; binary items often lack this sensitivity.
3. Complexity of suicide risk	Single item measures may fail to capture important determinants of suicide risk. Assessments should be comprehensive in capturing different aspects of ideation (incl. passive, active ideation, intent), and differentiate suicidal ideation from non-suicidal self-injurious thoughts.
4. Consider add-on ambulatory measures	Supplementing self-report EMA with ambulatory sensors (such as GPS and actigraphy) can provide objective data without increasing participant burden.
5. Optimize incentives	Monetary rewards are relatively uninfluential in increasing compliance rates; alternative personalized incentives (incl. receiving feedback on EMA responses) may be considered.
6. Ensure safety	Safety plans and clear guidelines on seeking help should always be implemented. Additional measures (e.g., ongoing monitoring) may be necessary for certain populations (incl. adolescents).
**Reporting**	
7. Reporting of adverse events	Adverse events should be assessed and transparently reported so that potential reactivity and the efficacy of different safety procedures can be evaluated.
8. Established EMA items	Databases of established EMA items are lacking. Clear reporting on item formulation and psychometric properties is needed. Questions from traditional questionnaire measures may not directly translate to the purposes of EMA.
9. Data quality	Factors that may impact data quality and interpretation (incl. attrition, compliance, patterns of missing data) need adequate reporting.

#### EMA in Clinical Practice

While only one of the reviewed studies employed EMA to assess the effectiveness of an intervention (53), EMA also has broad potential in applicability in clinical practice (24). Beyond EMA interventions (116, 117), EMA assessments in themselves may serve a therapeutic purpose: feedback from participants indicates that EMA made them more reflective, introspective, and mindful of their experiences [see e.g., (88)]. Further, for patients experiencing (persistent) suicidal ideation, demonstrating that ideation is variable, and hence malleable, may provide relief. In accordance with the finding that suicidal ideation variability may serve as a potential marker for increased suicide risk, this characteristic of ideation may be an especially valuable target for EMA monitoring and/or interventions in clinical practice. First applications of using EMA in clinical practice to monitor and manage symptoms are already underway (87). The extensive nature of EMA data also allows for more opportunities for single-case data analysis that may be used to examine individual symptom profiles or identify person-specific triggers (118)–an important goal in the treatment of the very heterogeneous group of patients experiencing STBs (119). However, despite these considerable inter-individual differences, most studies reviewed here solely examined group-level associations, while in clinical practice, the focus is on individual patients (120). Hence, the precise utility of this methodology in clinical practice in relation to STBs remains to be established.

#### Digital Phenotyping

The prospect of digital phenotyping of suicidal ideators (such as identifying those with high/low variability) based on EMA data has been discussed by many [see e.g., (121, 122)], but so far implemented by few (50, 57, 70). EMA data has revealed notable inter-individual differences in suicide symptom profiles (57), highlighting the importance of identifying meaningful subtypes of suicidal ideators that could improve risk assessments and choice of treatment targeting specific symptom profiles. However, the network theory is subject to certain pitfalls that still need to be solved before it can be implemented in clinical practice (24, 123). Next steps in EMA research may also involve intensive longitudinal assessments over longer time periods (i.e., months) in order to more reliably establish such phenotypes. Further, determining the value of such phenotyping would require additional follow-up assessments connecting these symptom profiles to overt outcomes (i.e., suicide attempt, mortality) over time.

### Conclusions

Currently, sociodemographic and clinical risk-factors, such as a current mental health diagnosis or previous attempt history, are considered the best predictors of future suicidal behavior–“the best” in this instance indicating the best of the worst, with currently established longitudinal risk factors being no better than chance at differentiating between those at high vs. low risk (33). More recently, real-time methodologies have identified new potential targets for risk-detection, namely rapid changes in momentary affect, interpersonal experiences, and sleep (124). However, these observations still warrant replication. The use of EMA in suicide research has grown rapidly in the past years, and review of the literature suggests that the fluctuating nature of suicidal ideation makes it an especially suited target for EMA, which may provide unique insights into the temporal correlates and imminent warning signs of increased suicide risk. Retrospective reports can be unreliable, especially when individuals are asked to recall fleeting or highly variable experiences (61), but EMA may have increased sensitivity in detecting these momentary experiences. Meanwhile, it has been proposed that identifying instability in suicidal ideation offers promise in improving the detection of those most at risk of suicide (37, 38), and attempts have been made to create new categorizations of suicidal ideators based on real-time data (50). Such risk profiling may hence represent next steps not only in EMA research, but in the improved treatment of patients with suicidal ideation.

## Author Contributions

LK and NA conceptualized the article. LK conducted the initial literature search and wrote the first draft of the manuscript. NA checked the literature search and provided feedback. NA, WvdD, and HR contributed to subsequent versions of the manuscript. All authors have approved the final manuscript.

## Funding

Funding for this study was provided by the Netherlands Organisation for Scientific Research (N.W.O) Research Talent Grant 406.18.521. N.W.O. had no role in the study design, collection, analysis or interpretation of the data, writing the manuscript, or the decision to submit the article for publication.

## Conflict of Interest

The authors declare that the research was conducted in the absence of any commercial or financial relationships that could be construed as a potential conflict of interest.

## Publisher's Note

All claims expressed in this article are solely those of the authors and do not necessarily represent those of their affiliated organizations, or those of the publisher, the editors and the reviewers. Any product that may be evaluated in this article, or claim that may be made by its manufacturer, is not guaranteed or endorsed by the publisher.

## Footnotes

^1^Interpretation of correlation coefficients based on *r* = 0.50 indicating large, *r* = 0.30 medium, and *r* = 0.10 small correlations (94).
